# Chromatin Dynamics *in Vivo*: A Game of Musical Chairs

**DOI:** 10.3390/genes6030751

**Published:** 2015-08-07

**Authors:** Daniël P. Melters, Jonathan Nye, Haiqing Zhao, Yamini Dalal

**Affiliations:** 1 Chromatin Structure and Epigenetics Mechanisms Unit, Center for Cancer Research, National Cancer Institute, National Institutes of Health, 41 Library Drive, Bethesda, MD 20892, USA; E-Mails: daniel.melters@nih.gov (D.P.M.); jon.nye@nih.gov (J.N.); haiqing.zhao@nih.gov (H.Z.); 2 Biophysics Graduate Program, University of Maryland, College Park, MD 20742, USA

**Keywords:** histones, chromatin, CENP-A, H3.3, H2A.Z, macroH2A, chaperones

## Abstract

Histones are a major component of chromatin, the nucleoprotein complex fundamental to regulating transcription, facilitating cell division, and maintaining genome integrity in almost all eukaryotes. In addition to canonical, replication-dependent histones, replication-independent histone variants exist in most eukaryotes. In recent years, steady progress has been made in understanding how histone variants assemble, their involvement in development, mitosis, transcription, and genome repair. In this review, we will focus on the localization of the major histone variants H3.3, CENP-A, H2A.Z, and macroH2A, as well as how these variants have evolved, their structural differences, and their functional significance *in vivo*.

## 1. Introduction

A canonical nucleosome wraps ~147 bp of DNA and is comprised of two copies of four histone proteins: H3, H4, H2A, and H2B [1]. This basic unit is repeated and forms a 10-nm chromatin fiber, the higher order folding of which has been a recent subject of much debate [2,3,4,5]. This debate has led to an emerging concept that the chromatin fiber is likely highly plastic and tunable. Chromatin allows for tight regulation of transcription, facilitates faithful segregation of chromosomes during cell division, and prevents DNA damage [6,7]. The use of the artificial LacO/LacI systems in combination with GFP-tagged proteins and derived systems has proven to be instrumental in assessing chromatin remodeling and fiber dynamics (reviewed in [8,9]). Indeed, combining single-nucleosome imaging with Monte Carlo computer simulations has shown that nucleosome dynamics can drive chromatin accessibility [10]. Recently, super-resolution microscopy showed that *in vivo*, nucleosomes are grouped in discrete domains along the chromatin fiber, where each cluster of nucleosomes differs in size, arguing against the possibility of uniform folding across the genome [11,12,13,14]. Despite these advances, the forces which act upon nucleosomal arrays to create distinctive clusters, how such arrays are maintained, whether such folding is driven by homogeneity or heterogeneity in histone variant composition, and how discrete folded arrays influence chromosomal domains, all remain exciting unanswered questions in the field.

Besides the canonical histones H3.1, H4, H2A and H2B, variants of these histones exist *in vivo* (Table 1). Such variants are thought to encode specialized nucleosomes with altered DNA-histone interaction and unique post-translational modifications (PTMs). Histone variants not only differ in sequence from their canonical counterparts, the timing of their expression is also different. Whereas canonical histones are expressed and incorporated during S phase, histone variants are spatiotemporally uncoupled from the regulation imposed on canonical histones. Thus, the composition of chromatin can be dynamic, changing throughout the cell cycle. Histone variants play important roles during development (reviewed in [15]), and mis-regulation of histone variants have been linked to cancer as they potentially alter gene expression and introduce genomic instability.

**Figure 1:**
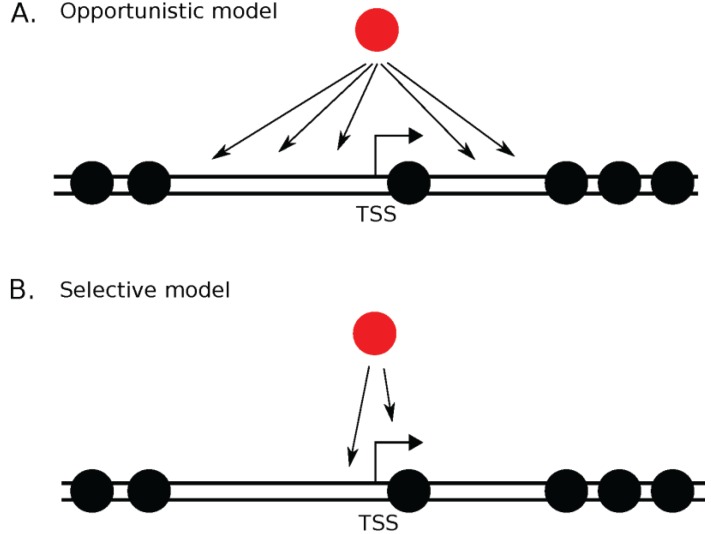
Are histone variants opportunistic occupiers or do they have exclusive targets? Replication-dependent histone variants (canonical H3.1, H3.2, H4, H2A, and H2B) are assembled into chromatin during S phase. On the other hand, replication-independent histone variants are assembled throughout the cell cycle (or late M/early G1 for CENP-A). Without the replication fork guiding the site of assembly, why do histone variants localize to sites where they are found? Two models exist. In the opportunistic model (**A**) histone variants are deposited in the chromatin once a nucleosome free region is presented, irrespective of the underlying DNA sequence or chromatin context. In the selective model (**B**) histone variants are deposited in either a sequence-specific manner, (such as a TATA box) via pre-bound transcription factors or associated transcription machinery, or through its chaperone. TSS = Transcription Start Site.

Consequently, understanding where and how histone variants are deposited into chromatin is an important biological question. The simplest possibility is that histone variants are opportunistic occupiers. An alternative possibility is that histone variants display spatiotemporal exclusivity occupying only specific loci, or at specific times during the cell cycle or gene activation. Recent data suggests that both models may be viable (Figure 1). As discussed below, depending on the spatiotemporal context, some histone variants can be deposited in an opportunistic fashion, or adhere to exclusive genomic loci. The same histone variant may behave differently depending on the presence or absence of preferred chaperones, or binding partners, and the number of molecules available relative to competitor histone. Recent data suggest that the dynamic interplay between histone variants and chaperones may act as a molecular rheostat, permitting the nucleus to fine-tune the genomic distribution of the histone variants not simply for long-term maintenance of domains, but for rapid response to stimuli. As we discuss later, perturbation of these states might shift this molecular rheostat of genome stability, contributing to disease or disease progression.

**Table 1:** Major histone variants in humans. For each major histone variant, the gene or gene clusters are reported, as well as its dependence on replication, its chaperone, distinctive functional features, and knock-out or knock-down phenotypes. * Genes encoding splice variants.

Histone	Genes	Replication	Chaperone	Function	Knockout/Knockdown Phenotype	Refs.
H2A	HIST2H2A (cluster)	independent, dependent	FACT, NAP-1	Canonical	N.D.	[1,15]
H2A.X	H2AFX	independent	FACT	Phosphorylated form marks ssDNA breaks	Genomic instability, growth retardation, immune deficiency, male infertility	[15,16,17]
macroH2A	H2AFY *, H2AFY2	independent	APLF	Contains macro domain, enriched on inactivated X chromosome	Impairs pre- and postnatal growth, interferes with reproductive efficiency	[15,18,19]
H2A.Z	H2AFZ *	independent	Tip60, SWR1	Contains acidic-patch, accumulation at +1 nucleosome of highly expressed genes	Embryonically lethal (E4.5–E7.5), impairs cellular proliferation, arrest in G1/S	[15,20,21,22,23,24,25,26,27]
H2A.B	H2AFB1, H2AFB2, H2AFB3	independent	NAP-1	Assoc. with active genes; strongly expressed in testis	Reduced efficiency in mRNA splicing,	[15,28,29,30,31]
H2B	H2BFM, H2BFS, H2BFWT, HIST2H2 (cluster)	independent, dependent	NAP-1	Canonical, monoubiquitinated form regulate transcription	N.D.	[1,15,32]
H3.1	HIST3H3 (cluster)	dependent	CAF-1, ASF1a, ASF1b	Canonical	N.D.	[1,15,32]
H3.2	HIST2H3C (cluster)	dependent	CAF-1, ASF1b	Canonical	N.D.	[15,32]
H3.3	H3F3A, H3F3B	independent	HIRA, ASF1a, ASF1b, DEK, ARTX/DAXX	Imprinted paternal genes; active genes, accumulation in senescent cells	infertility, genome instability, defective cell division and chromosome segregation	[15,32,33,34,35,36,37,38,39,40,41,42,43]
CENP-A	CENPA *	independent	HJURP, DAXX, RbAP46/48	Centromere-specific, incorporated in early G1	Chromosome missegregation; embryonically lethal	[15,44,45,46,47,48,49,50,51]
H4	HIST4H4 (cluster)	dependent	CAF-1	Canonical	N.D.	[1,15,32]

## 2. H3.3

**Figure 2:**
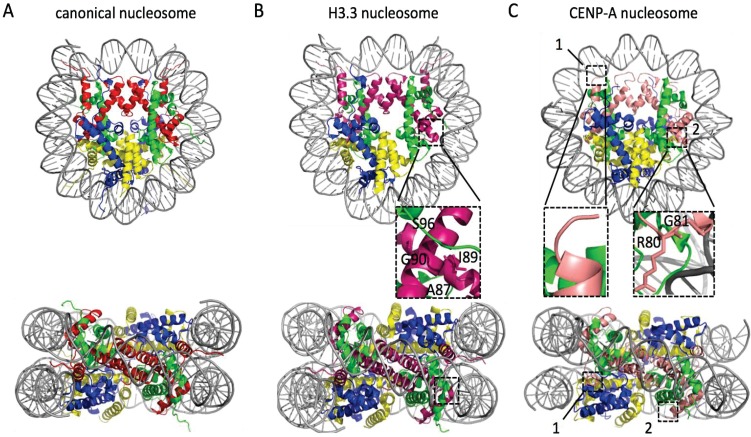

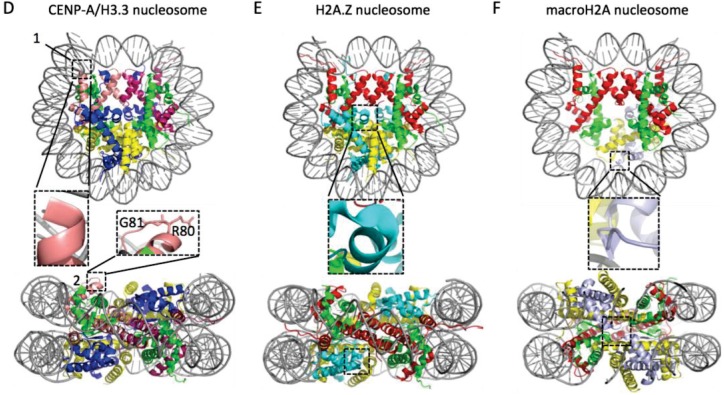
Crystal structures of nucleosomes containing histone variants. In each panel the nucleosome is shown in top and side view. In general, the colors used are red for H3, green for H4, blue for H2A, yellow for H2B, and grey for DNA. For the nucleosomes with histone variants (**B**–**F**) boxed areas and corresponding insets magnify specific differences between the canonical nucleosome (**A**) and the respective variant nucleosome. In (**B**) pink marks H3.3, a variant that differ from the canonical histone H3.1 by five amino acids, four of which are in alpha helix 2 of the histone fold domain and directly interact with H4 (box). These residues allow the CAF1, and HIRA and DAXX chaperones to discriminate between H3.1 and H3.3, respectively. To highlight these differences, one pair of H2A/H2B was peeled away from the top view. In (**C**) the centromere-specific CENP-A (in salmon) nucleosome is shown. This nucleosome wraps ~121 bp compared to ~147 in all other nucleosomes. This is due to a shorter alpha N helix (box 1 and 1') and a longer loop 1 (box 2 and 2'). In (**D**) the heterotypic CENP-A/H3.3 nucleosome is shown using the respective colors for CENP-A and H3.3. This heterotypic nucleosome displays the independent structural characteristics unique to the two histone variants, where box **D1** and **D2** correspond to **C1** and **C2** respectively, and wraps DNA with a bimodal length distribution: 133 bp or canonical 147 bp. In (**E**) the structure of H2A.Z nucleosome is shown, where H2A.Z is colored cyan. Despite only having 60% sequence similarity to the canonical H2A, H2A.Z nucleosome is structurally almost identical to (**A**) and wraps ~147 bp. The extended acidic patch of H2A.Z, that is thought to be important for its unique functions (see text), is highlighted by a box. Finally, in (**F**), the macroH2A nucleosome is shown. Because the linker between the histone fold domain and the macro domain of macroH2A is too flexible, it could not be crystallized. Therefore, the macro domain and the histone fold domain were crystallized separately and here only the histone fold domain is shown. To highlight the flexible and hydrophobic loop1-loop1 interface with a box, one dimer of macroH2A/H2B is peeled away in the top view. The crystal structures were obtained from the RCSB Protein Data Bank using the following identification codes: (**A**) 1AOI; (**B**) 3AV2; (**C**) 3AN2; (**D**) 3WTP; (**E**) 1F66; and (**F**) 1U35 and visualized using PyMOL software version 1.7.6.0 (Schrödinger, Cambridge, MD, USA, 2015).

Despite divergent amino-acid sequences, the overall structures of nucleosomes containing histone variants are surprisingly similar to canonical nucleosomes (Figure 2). For instance, histone variant H3.3 only differs by five amino acids from the canonical H3.1, and their crystal structures are super-imposable (Figure 2A,B) [1,32,33]. Interestingly, H3.3-specific residues are located on the accessible surfaces of H3/H4 tetramer, thus potentially selecting for H3.3-specific chaperones, rather than specifically altering the structure.

The two major histone H3 variants (H3.1 and H3.3) have been proposed to have evolved independently four times with H3.3 thought to be the ancestral H3 [34]. Interestingly, the amino-acid sequences of the two H3 variants are remarkably similar across the eukaryotic tree, emphasizing how stringent the purifying selection is on H3, including several PTM sites [34,35]. In many species, the derived H3.1 has become the canonical variant and is exclusively loaded during S phase [33], whereas H3.3 can be incorporated throughout the cell cycle, functioning as a replacement histone during transcription and DNA repair processes after depletion of H3.1 [36].

In mammals, H3.3 is encoded by two genes: H3F3A and H3F3B (Table 1). In mice, H3f3a mutants are viable to adulthood, although males show dysmorphic spermatozoa correlating with reduced male fertility, the basis of which is not fully understood [37]. H3f3a is expressed ubiquitously during mouse embryonic development until day E13.5, as well as adult heart, kidney, brain, testes, and ovaries [38]. In another series of experiments, a retroviral gene trap insertion of H3f3a created a hypomorphic mutation. The resulting mutant mice were indistinguishable from wild-type mice at birth, but nevertheless 50% died within 24 hours. Surviving mutant mice displayed retarded growth, impaired neuromuscular activity, and reduced fertility [38], pointing to the importance of the H3f3a in maintaining proper cellular activity. The phenotype for the H3f3b knockout was even equally severe, with 50% of H3f3b knock-out embryos dying during the second half of embryogenesis. Most of these embryos exhibited abnormal development indicative of a broad failure of embryonic growth [39]. An even more dramatic phenotype was observed when both H3.3s were knocked-down by morpholinos [40], or with siRNAs [40]. Morpholino disruption of H3.3 in *Xenopus* resulted in defects in late gastrulation, a phenotype mimicked by knock-down of the H3.3 chaperone HIRA (Table 1) [40]. Knock-down of both H3.3 genes in mouse oocytes resulted in arrest in early blastocyte stage. This phenotype is exclusively dependent on the maternal H3.3 pool to regulate the reactivation of imprinted genes in both the maternal and paternal genome [41], since the paternal genome has not yet been activated. Finally, a role for H3.3 in establishing heterochromatin at endogenous retroviral elements in mouse embryonic stem cells has been shown [42]. Altogether, these targeted gene disruption studies emphasize the importance of H3.3 in regulating various stages of development.

Independent of its importance in development, in slow dividing or non-replicative cells, H3.3 also accumulates at transcribed regions and sites of DNA repair [43]. Not only is H3.3 enriched at these genomic regions, it can also induce senescence together with its cleaved version (1–21 aa), which is incorporated into the chromatin by the chaperone HUCA complex, and subsequently represses the transcription of cell cycle regulators, presumably due to the loss of *N*-terminal modifications [43].

These observations above suggest that H3.3 can choose either the opportunistic or selective model (Figure 1). For example, in embryogenesis, retroviral elements and imprinted genes are specifically targeted by H3.3, and in senescent cells H3.3 targets cycle regulators. In contrast, H3.3 and its cleaved version can be incorporated at any transcribed region and site of DNA repair at the expense of H3.1 that was deposited during replication.

The quantity, genomic localization, and developmental timing of deposition of H3.3 are essential for a healthy cell. Any mis-regulation or mutation of H3.3 or its chaperones HIRA and DAXX (Table 1) could potentially lead to disease (reviewed in detail in [52]). Indeed, this is what is seen in solid pediatric high-grade gliomablastomas, chondrablastomas, and giant cell tumors of the bone (reviewed in [53,54]). A K27M mutation in H3.3 plays a dominant role in preventing the recruitment the polycomb complex [55,56,57,58]. On the other hand, mutations of H3.3 G34 are associated with global DNA hypomethylation and subsequent gene misregulation [59]. These two mutations are almost exclusive to the H3f3a genes, whereas the K36M mutation is predominantly found in the H3f3b gene [60]. K27M and G34R/V are mutually exclusive in tumors [59,61]. At the same time, knock-out of H3f3b results in ectopic CENP-A location [39]. This raises the intriguing possibility that histone H3 variants can co-opt a single ancestral pathway competitively, or cooperatively, leading to very different biological outcomes [62]. Thus, it is plausible that a regulatory link exists between each individual H3 variant and its respective chaperone, and this association might drive the choice of incorporation into specific genomic loci.

## 3. CENP-A

Where H3.1 and H3.3 only differ by five residues, the centromere-specific H3 variant CENP-A/cenH3 differs substantially, especially at the *N*- and *C*-terminus, α-N helix, and loop 1 region. These structural differences allow a CENP-A nucleosome to wrap only ~121 bp of DNA (Figure 2C), compared to ~147 bp for canonical nucleosomes [44]. In addition, the sequence of CENP-A loop 1 is hyper-variable and facilitates the rapid evolution of CENP-A [63,64], suppressing accumulation of selfish repetitive alpha satellite DNA, which could drive centromere expansion, resulting in unequal centromere strength during meiosis [63].

The paradoxically (paradoxical because CENP-A function is conserved) fast evolution of CENP-A has made it the most diverged H3 histone variant, which is commonly found throughout the eukaryotic kingdom, excluding holocentric insects [65] and some or all kinetoplastids (unicellular flagellated eukaryotes) [66,67]. The latter might not be surprising, since even kinetochore components in kinetoplastids are highly diverged from the other eukaryotes [68], suggesting they may have evolved a different mechanism to faithfully segregate chromosomes during cell division. Phylogenetic analyses of CENP-A variants have not produced a rooted tree [6,69,70], therefore it is unclear if CENP-A evolved once, or multiple times. Although CENP-A has an unclear evolutionary history, it does have a singularly well-defined function: it is the epigenetic mark of the centromere and is thought to encode unique structural features. Indeed, although this nucleosome has been extensively studied for nearly a decade, contradictory data suggest the existence of more than one nucleosomal conformation *in vivo* [48,61,71,72,73,74,75]. Even with purified components *in vitro*, CENP-A nucleosomes have been reported to display diverse behaviors, with budding yeast CENP-A being able to form stable octamers [76], unstable octamers [77], and hemisomal particles [78]. Similarly, the human CENP-A nucleosome can form relatively standard nucleosomal octamers [44,73,79], whereas other reports suggest a compacted octamer based on a rigid CENP-A/H4 tetrameric core [80,81]. Thus, using existing experimental tools, the CENP-A nucleosome displays complex dynamics. Therefore, definitive experimental confirmation of the different models will be required. Despite potential structural diversity of CENP-A’s nucleosomal forms, its centromeric localization is guided by an exclusive class of chaperones, such as HJURP in human cells [45,82], and is regulated in a cell-cycle specific manner [46,47,72,83,84,85,86,87,88], as well as by a cell-cycle regulated transcription of centromeres [89,90].

In contrast to centromeric CENP-A, CENP-A can also be targeted to ectopically incorporated lacO arrays [91,92,93]. In addition, artificially tagged and overexpressed CENP-A localizes ectopically to CTCF and transcription factor binding sites as a heterotypic CENP-A/H3.3 nucleosome [48]. Under wild type conditions, these sites are normally occupied by the transcriptionally coupled hybrid H3.3/H2A.Z nucleosomes [48], which are unstable, and undergo high histone turnover [94]. In addition, in human cancer cells ectopically localized CENP-A nucleosomes are also found to be enriched at DNase I hypersensitive sites, transcription factor binding sites, and potential enhancers [62]. Thus, an emerging concept is that ectopic CENP-A assembly is linked to histone turnover, potentially driven by remodelers or the act of transcription [89,90]. In the holocentric nematode *Caernohabditis elegans*, it was thought that CENP-A localization negatively correlates with regions transcribed in the germline [95]. Yet, recent analysis indicates that CENP-A nucleosomes localize to transcription factor binding hot spots in nematodes, which become occupied by transcription factors upon eviction of the CENP-A nucleosome [96]. Conversely, in human cell lines, it has been proposed that H3.3 serves as a placeholder for CENP-A during S phase when CENP-A is diluted because CENP-A nucleosomes are assembled only during early G1 [97]. Taken together, these data suggest an intricate interplay exists between transcriptional processes, CENP-A assembly, with an unexpected competition with H3.3 and transcription factor binding for the same DNA loci. The mechanism controlling the effect of transcriptional dynamics on CENP-A localization, at centromeres, or at ectopic loci, are key questions for future studies, especially focusing on the ability of ectopic CENP-A to promote or block gene regulation.

Natural duplication of CENP-A genes has also been found in some plants and animals. In the holocentric nematode *C. elegans* the duplicated CENP-A has adopted a meiosis-specific function [98], whereas in legume species multiple copies of CENP-A correlate with multiple centromeric CENP-A foci [99,100]. Coupled to artificial overexpressed CENP-A, which results in ectopic or non-centromeric CENP-A localization [48,62], overexpression of its chaperone HJURP also correlates with disease prognosis in gliomas [101,102,103,104], although it is unknown if overexpressed HJURP drives ectopic CENP-A localization. Finally, GFP-tagging tailswap CENP-A (H3 *N*-terminal tail attached to CENP-A histone fold domain) can lead to chromosome shattering in plants, a common feature of aggressive cancer in humans [105]. Overall, these observations argue for a model where CENP-A is selectively incorporated at the centromere, yet can spread beyond its centromere boundaries upon over-expression or mis-regulation.

To maintain CENP-A at the centromere, interactions with other CCAN proteins (constitutive centromere associated network) [106] are thought to be critical. One such protein is CENP-B, the only known centromeric protein to bind DNA in a sequence-specific manner. Recently it was shown that CENP-B forms a stable complex with CENP-A nucleosomes, in part through CENP-C [49,107], even though CENP-B knock-out mouse models only have mild phenotypes [108,109,110,111,112]. Although CENP-B binds the *N*-terminal tail of CENP-A [49], it is unknown if specific PTMs direct or preclude this binding. Whereas PTMs of H3 are conserved, and strongly associated with various chromatin states [15], it is surprising that the *N*-terminal tail of CENP-A is extremely fast evolving, showing plasticity in both length and sequence composition [63,69,70]. Progress has been made in this area of CENP-A research, and several PTMs have been reported for CENP-A [46,47,72,82,83,84,85,86,87,88]. For example, phosphorylation of S68 and mono-ubiquitinylation of K124 are required for CENP-A incorporation through HJURP [46,47]. In addition, phosphorylation of S16 and S18 are important for faithful mitotic chromosome segregation [84]. Similar modifications have also been found in budding yeast [113,114,115,116]. While the lack of sequence conservation of the CENP-A *N*-terminal tail makes it difficult to identify universal PTMs, a critical role for PTMs in regulating CENP-A chromatin throughout the cell cycle has become apparent and will undoubtedly be addressed in future studies.

## 4. Do CENP-A and H3.3 Compete for Chaperones?

As discussed above, ectopic CENP-A has been shown to form heterotypic CENP-A/H3.3 nucleosomes. The genomic loci where CENP-A/H3.3 nucleosomes are found are marked by active chromatin and high histone turnover [48], a property that was shown in parallel to be conserved in normal and cancer human cell lines [62]. Excitingly, the crystal structure of this heterotypic nucleosome was recently resolved [117], which showed that the physical properties of both CENP-A and H3.3 were retained in the hybrid nucleosome (Figure 2D). This nucleosome is thus inherently asymmetrical, with the two halves behaving independently of each other. Intriguingly, the hybrid nucleosome is thermally more stable than a homotypic CENP-A nucleosome, yet retains its capability to bind CENP-C *in vitro* [114]. An important implication of CENP-C binding is that CENP-A/H3.3 nucleosomes could conceivably attempt to form a kinetochore, with the key distinction being any resulting kinetochore would presumably assume an asymmetric state. Thus, the type of chromatin fiber formed, and consequent folding of such CENP-A/H3.3 arrays remains mysterious, as does altered stability at potential neocentromeres, and whether such neocentromeres would be weaker or more resilient than conventional centromeres. Another outstanding question is whether genomic sequence context could influence kinetochore formation on short or long arrays of CENP-A/H3.3 nucleosomes. The epigenetic inheritance of such arrays, with H3.1 assembled at S phase, H3.3 assembled throughout the cell cycle, and CENP-A normally assembled only in early G1, remains an exiting avenue of investigation. All of these new properties could potentially impact spindle attachments and response to tension. Finally, while the physiological role of arrays of CENP-A/H3.3 nucleosome remains unknown, it is plausible that within single CENP-A/H3.3 nucleosomes found at promoters and DNase I hypersensitive sites, increased external stability coupled to asymmetry in PTM information, might moderately impact transcriptional regulation of the underlying loci, driving competition with transcription factors and H3.3/H2A.Z nucleosomes.

For H3.3, two separate chaperone complexes are known to be responsible for it’s deposition. HIRA is a major and critically important H3.3 chaperone, incorporating H3.3 in a replication-independent manner into actively transcribed genes [118,119]. Meanwhile, ATRX/DAXX deposits H3.3 at telomeric and pericentric heterochromatin [118], as well as interstitial heterochromatin, regulating imprinted alleles in embryonic stem cells [119]. Furthermore, HIRA-dependent H3.3 deposition at developmentally important promoters is required for H3K27me3 and subsequent recruitment of components of the polycomb complex [120]. Surprisingly, CENP-A can incorporate ectopically whilst bound to ATRX/DAXX, possibly as part of the previously reported hybrid H3.3 nucleosome [48,62], as well as using RbAp48/p55 complexes when phosphorylated at S68 [46,51].

**Figure 3:**
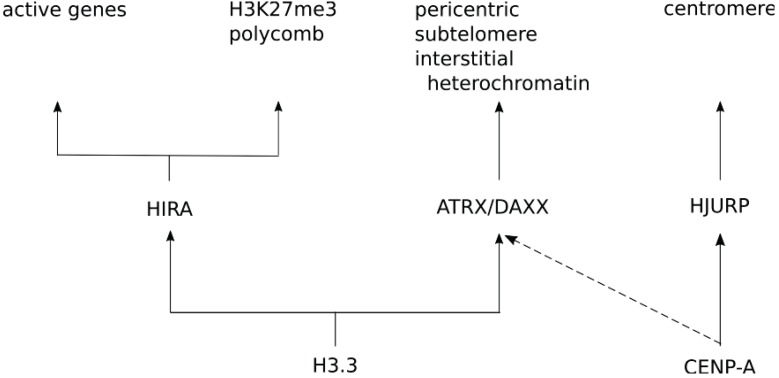
Competition between H3.3 and CENP-A for chaperone complex ATRX/DAXX. Normally CENP-A is directed to the centromere by its dedicated chaperone HJURP via a tightly regulated pathway (see text), whereas H3.3 is incorporated in various locations on the genome by its chaperones HIRA and ATRX/DAXX. HIRA directs H3.3 to chromatin and is required for establishing H3K27me3 at promoters of developmentally regulated genes in embryonic stem cells via the polycomb complex [120] as well as active genes [121,122]. On the other hand, ATRX/DAXX directs H3.3 to pericentric, subtelomeric, and interstitial heterochromatin [33,119]. In mice, H3.3 is encoded by two genes (H3f3a and H3f3b). Knock-out of H3f3b results in ectopic localization of CENP-A [39]. Overexpression of CENP-A also results in ectopic localization in human cell lines. In the latter case CENP-A is predominantly incorporated as a heterotypic CENP-A/H3.3 nucleosome via ATRX/DAXX chaperones [48]. These observations argue for a model where each gene product goes to specific sites in the genomes based on its association with each specific chaperone.

These results above may explain a recent observation that knocking out of one of the H3.3 genes (H3f3b) in mice [39] also resulted in ectopic CENP-A localization. In H3f3b knock-out MEFs chromosomes frequently mis-segregate and the ectopic CENP-A nucleosomes are thought to contribute by forming ectopic kinetochores [39]. We speculate that perhaps, both H3.3 and CENP-A compete for DAXX, but under normal stoichiometry DAXX preferentially binds H3.3/H4 because of its greater affinity thereby outcompeting CENP-A qualitatively and qualitatively (Figure 3). Under conditions where H3.3 (H3f3b) is depleted or CENP-A is overexpressed, DAXX may bind CENP-A and facilitates its ectopic incorporation as both homotypic CENP-A/CENP-A nucleosomes and heterotypic CENP-A/H3.3 nucleosomes. The implication of competition, or unwitting cooperation, between histone variants and chaperones is thus an unexplored yet highly promising avenue in chromosome cancer biology.

## 5. H2A.Z

Besides the various H3 variants, H2A also has histone variants. H2A.Z is only ~60% similar to H2A [20] and replaces H2A at sites flanking promoters of genes. H2A.Z nucleosomes exhibit rapid, replication-independent turnover [123]. Compared to canonical nucleosomes, H2A.Z nucleosomes is structurally very similar, albeit less stable at the interaction between the H2A.Z/H2B dimer and (H3/H4)_2_ tetramer [21]. The acidic patch on the surface of the nucleosome contains two additional residues (Figure 2E), which make contact with the H4 tail from neighboring nucleosomes [22]. The H2A.Z acidic patch also stimulates remodeling activity with the ISWI ATP-dependent remodeler [23,123]. Through this stimulation, H2A.Z is physically implicated in transcription, DNA repair, chromosome cohesion, centromere structure, and in maintaining heterochromatin and pericentric boundaries [24,25,26,124,125]. Finally, in it’s acetylated form, H2A.Z is predominantly found around transcription start sites [126,127], coupled with H3.3 containing acetylated K122. Cumulatively, it is thought that H3.3/H2A.Z hybrid nucleosomes are intrinsically unstable, which may facilitate rapid nucleosome eviction [90,128,129].

H2A.Z is commonly found across the eukaryotic domain (with the exception of dinoflagellates) and limited phylogenetic analyses suggest a single evolutionary origin [6,130]. Yet, in some species, a single H2A variant performs the role of H2A.X and H2A.Z, such as H2A.V in *Drosophila melanogaster* [22,23]. Nevertheless, H2A.Z is essential in *Tetrahymena*, fruit flies, and mice [131,132,133,134,135]. Unlike H2A, H2A.Z is constitutively expressed throughout the cell cycle, however it only makes up a small portion (~10%) of the total H2A pool in the cell [136]. Despite its relatively low abundance, its importance in various cellular processes has been shown [24,25,26,124,125].

Because of its relative low abundance, precise positioning of H2A.Z is expected, and indeed positioning of H2A.Z nucleosomes is a highly regulated process and recent work has shed light on multiple mechanisms that cooperate to ensure proper positioning of this important variant. For many years, it has been known that H2A.Z is enriched at eukaryotic promoters, specifically at the +1 and −1 nucleosomes flanking the nucleosome free regions associated with RNA pol II-transcribed genes [137,138,139]. H2A.Z incorporation at sites flanking the nucleosome free region relies on the conserved Swr1 complex. How targeting specificity was achieved was unknown until recent work showed that, *in vitro,* the Swr1 complex specifically binds to long nucleosome-free DNA commonly found at gene promoters [140]. Interestingly, the Swr1 complex subunit Swc2 may play multiple roles in H2A.Z regulation by serving as the DNA binding component targeting Swr1 to nucleosome free regions as well as binding H2A.Z nucleosomes and acting as a molecular lock that prevents its exchange for H2A [141]. In this way, H2A.Z can be maintained at nucleosomes flanking the nucleosome free region at promoters and allow for proper regulation of transcription. Recent work in *Drosophila* has shown that the presence of H2A.Z at the +1 nucleosome can reduce RNA pol II stalling by decreasing the barrier to transcription progression, thus facilitating gene expression [142].

In addition to the mechanisms regulating deposition of H2A.Z in the genome, new data has uncovered a number of pathways responsible for catalyzing the exchange of H2A.Z for H2A. In contrast to the Swr1 complex’s well-known role in depositing H2A.Z, evidence suggests that H3K56Ac within the H2A.Z nucleosome can lead to Swr1 mediated removal of H2A.Z [141]. Furthermore, the human ANP32E protein, a member of the P400/TIP-60 histone exchange complex, has been implicated in the selective removal of H2A.Z from the transcription start site and enhancer regions [143]. In budding yeast the activity of the H2A chaperones FACT and Spt6 are necessary to prevent mis-incorporation of H2A.Z within gene bodies, suggesting that depletion of canonical histones may lead to promiscuous incorporation of H2A.Z, since the Swr1 complex favors nucleosome free regions [144]. Thus the incorporation of H2A.Z into specific regions of chromatin is tightly regulated, providing strong evidence for the “selective model” (Figure 1).

The localization of H2A.Z could be dynamic, changing throughout the cell cycle. For instance, in mouse trophoblast stem cells, H2A.Z is lost from gene promoters and relocalizes to the centromere upon entry into mitosis [145]. Once at the centromere, it is involved in organizing the 3D structure of centromeric chromatin and has been shown to form distinct domains containing either H3K4me2 or H3K9me3 [25]. Unlike mammalian cells, H2A.Z in fission yeast is excluded from centromeric chromatin and is thought to have a role in preventing stable incorporation of the CENP-A at ectopic sites [27,146]. Interestingly, even though its role may differ in these organisms, knockdown of H2A.Z leads to defects in chromosome segregation including lagging chromosomes and chromosome bridges in both mice and fission yeast [147,148]. However, in budding yeast acetylation of H2A.Z is essential for cohesion of sister chromatids during mitosis [125]. Thus, the mechanisms controlling the dynamic localization of H2A.Z throughout the cell cycle and its precise role at the centromere during mitosis are key questions to be addressed in future studies.

## 6. macroH2A

Whereas H2A.Z is associated with active transcription, macroH2A was initially discovered on the inactivated X chromosome in female cells [149]. MacroH2A is characterized by the presence of a large (~30 kDa) *C*-terminal macro domain connected to H2A-like histone fold domain via a short, flexible linker that protrudes from the nucleosome core [150]. The primary structural difference between canonical nucleosome and macroH2A nucleosomes (Figure 2F) resides in the loop 1-loop 1 interface, which is less flexible and more hydrophobic. These structural features support the correlation between macroH2A and silenced chromatin. In addition, HDACs physically interact with the macro domain of macroH2A [150]. A hallmark of silenced chromatin is limited turnover rate of nucleosomes [151]; it is therefore possible that macroH2A’s half-life is long compared to canonical H2A, creating a stably incorporated nucleosome facilitating silenced chromatin.

Phylogenetic distribution of macroH2A is non-uniform, as macroH2A is found in some basal metazoan species and craniates, but missing in insects, nematodes, and tunicates [69]. A H2A variant functionally similar to macroH2A, albeit without the macro domain, might exist in plants as well [152]. In mice and humans there are two genes that encode for macroH2A: macroH2A1 and macroH2A2, with macroH2A1 being the most abundant variant. In addition, macroH2A1 has two isoforms, differing by the alternative use of a single exon, creating two isoforms with distinct functional differences. Only macroH2A1.1 can bind polymeric and monomeric ADP-ribose as well as *O*-acetyl ADP-ribose [153,154] and is regulated by PARP1 [155,156]. Through the recruitment of PARP1, macroH2A1.1 promotes the CEBP-mediated acetylation of H2B at residues K12 and K120, either positively or negatively regulating the expression of macroH2A1-target genes [157]. Furthermore, in a recent study, chromatin immunoprecipitation coupled with transcriptional profiling showed that macroH2A1 occupies promoters of both expressed and repressed genes [158]. Puzzlingly, at expressed genes, macroH2A1 masks repressor-binding sites, whereas at repressed genes macroH2A masks activator-binding sites. How macroH2A can enact dichotomous functions remains to be determined. The chaperone depositing macroH2A at the X chromosome, the timing of macroH2A assembly, the remodeler which effects its removal, and whether macroH2A can be stably inherited after replication, all remain unsolved mysteries.

One clue comes from macroH2A localization at senescence-associated heterochromatin foci, where HIRA and ASF1a [159] appear to deposit macroH2A. As noted previously, HIRA and ASF1a are also responsible for H3.3 deposition at actively transcribed genes [118,119]. Thus, whether these chaperones are bona fide macroH2A chaperones or represent a situation similar to CENP-A (which exploits DAXX under specific conditions) remains to be determined. Nevertheless, macroH2A is not an essential histone variant, because a double knock-out of both macroH2A genes in mice does not result in death, despite impaired pre- and postnatal growth in addition to male reproductive impairments [18]. Altogether, the role of macroH2A in heterochromatin formation and enhancer regulation might be important, yet redundant, with alternative mechanisms contributing to similar functions.

Finally, in cancer cells, a switch in the expression from PARP1-regulated macroH2A1.1 to PARP1-insensitive macroH2A1.2 is observed [160,161]. Indeed, restoration of macroH2A1.1 expression in melanoma cell lines limits the proliferation capacity of malignant melanomas [162]. MacroH2A1.1 is also a target for PARP1, which in turn is a common target for cancer drug development, because of PARP-1’s involvement in many cellular activities including DNA repair and transcription factor regulation [163,164,165]. By removing macroH2A1.1, cancer cells transform their macroH2A1.2-associated silenced chromatin to a chromatin state more similar to the inactivated X chromosome. Thus, it is imperative to figure out how a cell switches from one macroH2A isoform to another for developing potential future cancer treatments focusing on histone variants.

## 7. Evolution of Histone Variants

Dramatic chromatin rearrangements and mis-regulation of histone variants [61,166,167] are a feature of cancer cells [168]. Mis-regulated histone variants, resulting in increased quantity of its gene product, might be incorporated through an ancestral assembly pathway, facilitating the cancer phenotype. In addition, unique solutions for generating specific chromatin states, for instance, how holocentric insects create functional kinetochore in the absence of CENP-A and CENP-C [64], might provide insights into innovative pathways that have evolved to deal with the same mechanical and structural problems involved in chromosome segregation.

It is therefore of interest to understand when histone variants and their chaperones arose during evolution. The vast majority of eukaryotes wrap their DNA around nucleosomes, except for dinoflagellates, which wrap their genomes loosely around histone-like protein of bacterial origin [169,170,171]. It is thought that the building blocks of nucleosomes predate the eukaryotic domain [34,35,172], as archaea have histone-like proteins called Hmfs. These Hmfs are structurally similar to histone proteins [173] and wrap ~70 bp of DNA in a right-handed and left-handed tetrameric nucleosome [174], but lack a *N*- or *C*-terminal tail [175], characteristic for eukaryotic histone proteins. Nevertheless, all known eukaryotic histones have *N*-terminal tails and some histone PTMs are thought to be conserved [34] arguing for the coevolution of histone modifying enzymes and histones. In addition, another constraining factor in histone evolution could be the co-evolution of histones with transcription factors (reviewed in [176]) and with their chaperones [177], potentially facilitating the evolution of an intricate framework for regulation of gene transcription and specialized chromatin states. How histone variants and their regulatory pathways have evolved is relevant for understanding how cancer cells can escape mis-regulation by mutations in histones, their variants, and their chaperones.

## 8. Conclusions and Future Perspectives

Why and how do histone variants localize to sites where they are found? Here, we describe the emergence of a complex picture in which some histone variants are opportunistic and localize where space becomes available (for example, H3.3 in transcribed regions and senescent cells), whereas other variants are primarily limited to a specific locus (for example, CENP-A localization to the centromere and H2A.Z at the −1 and +1 nucleosome around the transcription start site) (Figure 1). In the case of CENP-A, its selective localization seems to be determined, at least in part, by its unique chaperone HJURP. Yet, when one of the H3.3 genes is knocked out or CENP-A is overexpressed, CENP-A effortlessly occupies ectopic sites. Furthermore, mis-regulation of any of the histone variants and their respective chaperones is correlated with cancer progression. Many questions remain unanswered. First, how are histone chaperones regulated? Second, is there competition between histone variants for specific chaperones driven by relative affinities? Third, is there a role for histone variants in terminally differentiated cells, as documented in a recent study in mice which showed a role for the exchange of H2A.Z from promoter regions of neural genes involved in memory consolidation of fear [178]. Fourth, how are histone variants specifically removed from and re-incorporated in chromatin? Fifth, although most of genomic DNA exists in its B configuration, other non-B DNA structures exist, yet little is known about any association with specific histone variants. Do histone variants facilitate the stabilization of non-B-DNA structures [179]? Does mis-regulation or mutation of histone variants simply correlate with, or mechanistically cause or accelerate human disease [179,180]? Finally, the evolution of chromatin coincides with the evolution of transcriptional regulation (reviewed in [176]). Deciphering how this co-evolution created the various histone assembly pathways will provide important evolutionary insight in how chromatin has evolved as a whole, and reveal the constraints under which histone variants and their respective chaperones function. Answering all these questions will contribute to a more complete conceptual framework of how the genome is regulated.

## References

[1] Luger K., Mader A.W., Richmond R.K., Sargent D.F., Richmond T.J. (1997). Crystal structure of the nucleosome core particle at 2.8 A resolution. Nature.

[2] Efroni S., Carmel L., Schaefer C.G., Buetow K.H. (2008). Superposition of transcriptional behaviors determines gene state. PLoS ONE.

[3] Fussner E., Strauss M., Djuric U., Li R., Ahmed K., Hart M., Ellis J., Bazett-Jones D.P. (2012). Open and closed domains in the mouse genome are configured as 10-nm chromatin fibres. EMBO Rep..

[4] Joti Y., Hikima T., Nishino Y., Kamada F., Hihara S., Takata H., Ishikawa T., Maeshima K. (2012). Chromosomes without a 30-nm chromatin fiber. Nucleus.

[5] Quénet D., McNally J.G., Dalal Y. (2012). Through thick and thin: The conundrum of chromatin fibre folding *in vivo*. EMBO Rep..

[6] Malik H.S., Henikoff S. (2003). Phylogenomics of the nucleosome. Nat. Struct. Biol..

[7] Rieder D., Trajanoski Z., McNally J.G. (2012). Transcription factories. Front. Genet..

[8] Belmont A.S. (2001). Visualizing chromosome dynamics with GFP. Trends Cell Biol..

[9] Dion V., Gasser S.M. (2013). Chromatin movement in the maintenance of genome stability. Cell.

[10] Hirara S., Pack C.G., Kaizu K., Tani T., Hanafusa T., Nozaki T., Takemoto S., Yoshimi T., Yokota H., Imamoto N. (2012). Local nucleosome dynamics facilitate chromatin accessibility in living mammalian cells. Cell Rep..

[11] Van Bortle K., Corces V.G. (2013). The role of chromatin insulators in nuclear architecture and genome function. Curr. Opin. Genet. Dev..

[12] Tai P.W., Zaidi S.K., Wu H., Grandy R.A., Montecino M., van Wijnen A.J., Lian J.B., Stein G.S., Stein J.L. (2014). The dynamic architectural and epigenetic nuclear landscape: Developing the genomic almanac of biology and disease. J. Cell. Physiol..

[13] Ricci M.A., Manzo C., García-Parajo M.F., Lakadamyali M., Cosma M.P. (2015). Chromatin fibers are formed by heterogeneous groups of nucleosomes *in vivo*. Cell.

[14] Cremer T., Cremer M., Hübner B., Strickfaden H., Smeets D., Popken J., Sterr M., Markaki Y., Rippe K., Cremer C. (2015). The 4D nucleome: Evidence for a dynamic nuclear landscape based on co-aligned active and inactive nuclear compartments. FEBS Lett..

[15] Maze I., Noh K.M., Soshnev A.A., Allis C.D. (2014). Every amino acid matters: Essential contributions of histone variants to mammalian development and disease. Nat. Rev. Genet..

[16] Celeste A., Petersen S., Romanienko P.J., Fernandez-Capetillo O., Chen H.T., Sedelnikova O.A., Reina-San-Martin B., Coppola V., Meffre E., Difilippantonio M.J. (2002). Genomic instability in mice lacking histone H2AX. Science.

[17] Heo K., Kim H., Choi S.H., Choi J., Kim K., Gu J., Lieber M.R., Yang A.S., An W. (2008). FACT-mediated exchange of histone variant H2AX regulated by phosphorylation of H2AX and ADP-ribosylation of Spt16. Mol. Cell.

[18] Pehrson J.R., Changolkar L.N., Costanzi C., and Leu N.A. (2014). Mice without MacroH2A Histone Variants. Mol. Cell. Biol..

[19] Mehrotra P.V., Ahel D., Ryan D.P., Weston R., Wiechens N., Kraehenbuehl R., Owen-Hughes T., Ahel I. (2011). DNA repair factor APLF is a histone chaperone. Mol. Cell.

[20] Zlatanova J., Thakar A. (2008). H2A.Z: View from the top. Structure.

[21] Suto R.K., Clarkson M.J., Tremethick D.J., Luger K. (2000). Crystal structure of a nucleosome core particle containing the variant histone H2A.Z. Nat. Struct. Biol..

[22] Kalashnikova A.A., Porter-Goff M.E., Muthurajan U.M., Luger K., Hansen J.C. (2013). The role of the nucleosome acidic patch in modulating higher order chromatin structure. J. R. Soc. Interface.

[23] Goldman J.A., Garlick J.D., Kingston R.E. (2010). Chromatin remodeling by imitation switch (ISWI) class ATP-dependent remodelers is stimulated by histone variant H2A.Z. J. Biol. Chem..

[24] Meneghini M.D., Wu M., Madhani H.D. (2003). Conserved histone variant H2A.Z protects euchromatin from the ectopic spread of silent heterochromatin. Cell.

[25] Greaves I.K., Rangasamy D., Ridgway P., Tremethick D.J. (2007). H2A.Z contributes to the unique 3D structure of the centromere. Proc. Natl. Acad. Sci. USA.

[26] Erdel F., Rippe K. (2011). Chromatin remodelling in mammalian cells by ISWI-type complexes—Where, when and why?. FEBS J..

[27] Hou H., Wang Y., Kallgren S.P., Thompson J., Yates J.R., Jia S. (2010). Histone variant H2A.Z regulates centromere silencing and chromosome segregation in fission yeast. J. Biol. Chem..

[28] Okuwaki M., Kato K., Shimahara H., Tate S., Nagata K. (2005). Assembly and disassembly of nucleosome core particles containing histone variants by human nucleosome assembly protein I. Mol. Cell Biol..

[29] González-Romero R., Méndez J., Ausió J., Eirín-López J.M. (2008). Quickly evolving histones, nucleosome stability and chromatin folding: All about histone H2A.Bbd. Gene.

[30] Arimura Y., Kimura H., Oda T., Sato K., Osakabe A., Tachiwana H., Sato Y., Kinugasa Y., Ikura T., Sugiyama M. (2013). Structural basis of a nucleosome containing histone H2A.B/H2A.Bbd that transiently associates with reorganized chromatin. Sci. Rep..

[31] Sansoni V., Casas-Delucchi C.S., Rajan M., Schmidt A., Bönisch C., Thomae A.W., Staege M.S., Hake S.B., Cardoso M.C., Imhof A. (2014). The histone variant H2A.Bbd is enriched at sites of DNA synthesis. Nucleic Acids Res..

[32] Tachiwana H., Osakabe A., Shiga T., Miya Y., Kimura H., Kagawa W., Kurumizaka H. (2011). Structures of human nucleosomes containing major histone H3 variants. Acta Crystallogr. D Biol. Crystallogr..

[33] Szenker E., Ray-Gallet D., Almouzni G. (2011). The double face of the histone variant H3.3. Cell Res..

[34] Waterborg J.H. (2012). Evolution of histone H3: Emergence of variants and conservation of post-translational modification sites. Biochem. Cell Biol..

[35] Postberg J., Forcob S., Chang W.J., Lipps H.J. (2010). The evolutionary history of histone H3 suggests a deep eukaryotic root of chromatin modifying mechanisms. BMC Evol. Biol..

[36] Ahmad K., Henikoff S. (2002). Epigenetic consequences of nucleosome dynamics. Cell.

[37] Tang M.C., Jacobs S.A., Mattiske D.M., Soh Y.M., Graham A.N., Tran A., Lim S.L., Hudson D.F., Kalitsis P., O’Bryan M.K. (2015). Contribution of the two genes encoding histone variant h3.3 to viability and fertility in mice. PLoS Genet..

[38] Couldrey C., Carlton M.B., Nolan P.M., Colledge W.H., Evans M.J. (1999). A retroviral gene trap insertion into the histone 3.3A gene causes partial neonatal lethality, stunted growth, neuromuscular deficits and male sub-fertility in transgenic mice. Hum. Mol. Genet..

[39] Bush K.M., Yuen B.T., Barrilleaux B.L., Riggs J.W., O’Geen H., Cotterman R.F., Knoepfler P.S. (2013). Endogenous mammalian histone H3.3 exhibits chromatin-related functions during development. Epigenetics Chromatin.

[40] Szenker E., Lacoste N., Almouzni G. (2012). A developmental requirement for HIRA-dependent H3.3 deposition revealed at gastrulation in *Xenopus*. Cell Rep..

[41] Wen D., Banaszynski L.A., Liu Y., Geng F., Noh K.M., Xiang J., Elemento O., Rosenwaks Z., Allis C.D., Rafii S. (2014). Histone variant H3.3 is an essential maternal factor for oocyte reprogramming. Proc. Natl. Acad. Sci. USA.

[42] Elsässer S.J., Noh K.M., Diaz N., Allis C.D., Banaszynski L.A. (2015). Histone H3.3 is required for endogenous retroviral element silencing in embryonic stem cells. Nature.

[43] Duarte L.F., Young A.R., Wang Z., Wu H.A., Panda T., Kou Y., Kapoor A., Hasson D., Mills N.R., Ma’ayan A. (2014). Histone H3.3 and its proteolytically processed form drive a cellular senescence programme. Nat. Commun..

[44] Tachiwana H., Kagawa W., Shiga T., Osakabe A., Miya Y., Saito K., Hayashi-Takanaka Y., Oda T., Sato M., Park S.Y. (2011). Crystal structure of the human centromeric nucleosome containing CENP-A. Nature.

[45] Foltz D.R., Jansen L.E., Bailey A.O., Yates J.R., Bassett E.A., Wood S., Black B.E., Cleveland D.W. (2009). Centromere-specific assembly of CENP-a nucleosomes is mediated by HJURP. Cell.

[46] Yu Z., Zhou X., Wang W., Deng W., Fang J., Hu H., Wang Z., Li S., Cui L., Shen J. (2015). Dynamic phosphorylation of CENP-A at Ser68 orchestrates its cell-cycle-dependent deposition at centromeres. Dev. Cell.

[47] Niikura Y., Kitagawa R., Ogi H., Abdulle R., Pagala V., Kitagawa K. (2015). CENP-A K124 Ubiquitylation is required for CENP-A deposition at the centromere. Dev. Cell.

[48] Lacoste N., Woolfe A., Tachiwana H., Garea A.V., Barth T., Cantaloube S., Kurumizaka H., Imhof A., Almouzni G. (2014). Mislocalization of the centromeric histone variant CenH3/CENP-A in human cells depends on the chaperone DAXX. Mol. Cell.

[49] Fachinetti D., Han J.S., McMahon M.A., Ly P., Abdullah A., Wong A.J., Cleveland D.W. (2015). DNA sequence-specific binding of CENP-B enhances the fidelity of human centromere function. Dev. Cell.

[50] Regnier V., Vagnarelli P., Fukagawa T., Zerjal T., Burns E., Trouche D., Earnshaw W., Brown W. (2005). CENP-A is required for accurate chromosome segregation and sustained kinetochore association of BubR1. Mol. Cell Biol..

[51] Furuyama T., Dalal Y., Henikoff S. (2006). Chaperone-mediated assembly of centromeric chromatin *in vitro*. Proc. Natl. Acad. Sci. USA.

[52] Filipescu D., Müller S., Almouzni G. (2014). Histone H3 variants and their chaperones during development and disease: Contributing to epigenetic control. Annu. Rev. Cell Dev. Biol..

[53] Kallappagoudar S., Yadav R.K., Lowe B.R., Partridge J.F. (2015). Histone H3 mutations—A special role for H3.3 in tumorigenesis?. Chromosoma.

[54] Appin C.L., Brat D.J. (2015). Molecular pathways in gliomagenesis and their relevance to neuropathologic diagnosis. Adv. Anat. Pathol..

[55] Bender S., Tang Y., Lindroth A.M., Hovestadt V., Jones D.T., Kool M., Zapatka M., Northcott P.A., Sturm D., Wang W. (2013). Reduced H3K27me3 and DNA hypomethylation are major drivers of gene expression in K27M mutant pediatric high-grade gliomas. Cancer Cell.

[56] Lewis P.W., Müller M.M., Koletsky M.S., Cordero F., Lin S., Banaszynski L.A., Garcia B.A., Muir T.W., Becher O.J., Allis C.D. (2013). Inhibition of PRC2 activity by a gain-of-function H3 mutation found in pediatric glioblastoma. Science.

[57] Venneti S., Felicella M.M., Coyne T., Phillips J.J., Gorovets D., Huse J.T., Kofler J., Lu C., Tihan T., Sullivan L.M. (2013). Histone 3 lysine 9 trimethylation is differentially associated with isocitrate dehydrogenase mutations in oligodendrogliomas and high-grade astrocytomas. J. Neuropathol. Exp. Neurol..

[58] Chan K.M., Fang D., Gan H., Hashizume R., Yu C., Schroeder M., Gupta N., Mueller S., James C.D., Jenkins R. (2013). The histone H3.3K27M mutation in pediatric glioma reprograms H3K27 methylation and gene expression. Genes Dev..

[59] Sturm D., Witt H., Hovestadt V., Khuong-Quang D.A., Jones D.T., Konermann C., Pfaff E., Tönjes M., Sill M., Bender S. (2012). Hotspot mutations in H3F3A and IDH1 define distinct epigenetic and biological subgroups of glioblastoma. Cancer Cell.

[60] Behjati S., Tarpey P.S., Presneau N., Scheipl S., Pillay N., van Loo P., Wedge D.C., Cooke S.L., Gundem G., Davies H. (2013). Distinct H3F3A and H3F3B driver mutations define chondroblastoma and giant cell tumor of bone. Nat. Genet..

[61] Schwartzentruber J., Korshunov A., Liu X.Y., Jones D.T., Pfaff E., Jacob K., Sturm D., Fontebasso A.M., Quang D.A., Tönjes M. (2012). Driver mutations in histone H3.3 and chromatin remodelling genes in paediatric glioblastoma. Nature.

[62] Athwal R.K., Walkiewicz M.P., Baek S., Fu S., Bui M., Camps J., Ried T., Sung M.H., Dalal Y. (2015). CENP-A nucleosomes localize to transcription factor hotspots and subtelomeric sites in human cancer cells. Epigenetics Chromatin.

[63] Henikoff S., Ahmad K., Malik H.S. (2001). The centromere paradox: Stable inheritance with rapidly evolving DNA. Science.

[64] Malik H.S., Henikoff S. (2009). Major evolutionary transitions in centromere complexity. Cell.

[65] Drinnenberg I.A., de Young D., Henikoff S., Malik H.S. (2014). Recurrent loss of CenH3 is associated with independent transitions to holocentricity in insects. Elife.

[66] Lowell J.E., Cross G.A. (2004). A variant histone H3 is enriched at telomeres in *Trypanosoma brucei*. J. Cell Sci..

[67] Berriman M., Ghedin E., Hertz-Fowler C., Blandin G., Renauld H., Bartholomeu D.C., Lennard N.J., Caler E., Hamlin N.E., Haas B. (2005). The genome of the African trypanosome *Trypanosoma brucei*. Science.

[68] Akiyoshi B., Gull K. (2014). Discovery of unconventional kinetochores in kinetoplastids. Cell.

[69] Talbert P.B., Ahmad K., Almouzni G., Ausió J., Berger F., Bhalla P.L., Bonner W.M., Cande W.Z., Chadwick B.P., Chan S.W. (2012). A unified phylogeny-based nomenclature for histone variants. Epigenetics Chromatin.

[70] Maheshwari S., Tan E.H., West A., Franklin F.C., Comai L., Chan S.W. (2015). Naturally occurring differences in CENH3 affect chromosome segregation in zygotic mitosis of hybrids. PLoS Genet..

[71] Black B.E., Cleveland D.W. (2011). Epigenetic centromere propagation and the nature of CENP-A nucleosomes. Cell.

[72] Bui M., Dimitriadis E.K., Hoischen C., An E., Quénet D., Giebe S., Nita-Lazar A., Diekmann S., Dalal Y. (2012). Cell-cycle-dependent structural transitions in the human CENP-A nucleosome *in vivo*. Cell.

[73] Hasson D., Panchenko T., Salimian K.J., Salman M.U., Sekulic N., Alonso A., Warburton P.E., Black B.E. (2013). The octamer is the major form of CENP-A nucleosomes at human centromeres. Nat. Struct. Mol. Biol..

[74] Walkiewicz M.P., Dimitriadis E.K., Dalal Y. (2014). CENP-A octamers do not confer a reduction in nucleosome height by AFM. Nat. Struct. Mol. Biol..

[75] Henikoff S., Ramachandran S., Krassovsky K., Bryson T.D., Codomo C.A., Brogaard K., Widom J., Wang J.P., Henikoff J.G. (2014). The budding yeast Centromere DNA Element II wraps a stable Cse4 hemisome in either orientation *in vivo*. Elife.

[76] Kingston I.J., Yung J.S., Singleton M.R. (2011). Biophysical characertization of the centromere-specific nulceosome from budding yeast. J. Biol. Chem..

[77] Dechasse M.L., Wyns K., Li M., Hall M.A., Wang M.D., Luger K. (2011). Structure of Scm3-mediated assembly of budding yeast centromeric nucleosomes. Nat. Commun..

[78] Furuyama T., Codomo C.A., Henikoff S. (2013). Reconstitution of hemisomes on budding yeast centromeric DNA. Nucleic Acid Res..

[79] Yoda K., Ando S., Morishita S., Houmura K., Hashimoto K., Takeyasu K., Okazaki T. (2000). Human centromere protein A (CENP-A) can replace histone H3 in nucleosome reconstitution *in vitro*. Proc. Natl Acad. Sci. U.S.A..

[80] Sekulic N., Bassett E.A., Rogers D.J., Black D.E. (2010). The structure of (CENP-A-H4)(2) reveals physical features that mark centromeres. Nature.

[81] Miell M.D., Fuller C.J., Guse A., Barysz H.M., Downes A., Owen-Hughes T., Rappsilber T., Straight A.F., Allshire R.C. (2013). CENP-A confers a reduction in height on octameric nucleosomes. Nat. Struct. Mol. Biol..

[82] Dunleavy E.M., Roche D., Tagami H., Lacoste N., Ray-Gallet D., Nakamura Y., Daigo Y., Nakatani Y., Amounzi-Pettinotti G. (2009). HJURP is a cell-cycle-dependent maintenance and deposition factor of CENP-A at centromeres. Cell.

[83] Wisniewski J., Hajj B., Chen J., Mizuguchi G., Xiao H., Wei D., Dahan M., Wu C. (2014). Imaging the fate of histone Cse4 reveals *de novo* replacement in S phase and subsequent stable residence at centromeres. Elife.

[84] Schuh M., Lehner C.F., Heidmann S. (2007). Incorporation of Drosophila CID/CENP-A and CENP-C into centromeres during early embryonic anaphase. Curr. Biol..

[85] Jansen L.E., Black B.E., Foltz D.R., Cleveland D.W. (2007). Propagation of centromeric chromatin requires exit from mitosis. J. Cell Biol..

[86] Silva M.C., Bodor D.L., Stellfox M.E., Martins N.M., Hochegger H., Foltz D.R., Jansen L.E. (2012). Cdk activity couples epigenetic centromere inheritance to cell-cycle progression. Dev. Cell.

[87] Bailey A.O., Panchenko T., Sathyan K.M., Petkowski J.J., Pai P.J., Bai D.L., Russell D.H., Macara I.G., Shabanowitz J., Hunt D.F. (2013). Posttranslational modification of CENP-A influences the conformation of centromeric chromatin. Proc. Natl. Acad. Sci. USA.

[88] Zeitlin S.G., Shebly R.D., Sullivan K.F. (2001). CENP-A is phosphorylated by Aurora B kinase and plays an unexpected role in completion of cytokinesis. J. Cell Biol..

[89] Chan F.L., Marshall O.J., Saffery R., Kim B.W., Earle E., Choo K.H., Wong L.H. (2012). Active transcription and essential role of RNA polymerase II at the centromere during mitosis. Proc. Natl. Acad. Sci. USA.

[90] Quénet D., Dalal Y. (2014). A long non-coding RNA is required for targeting centromeric protein A to the human centromere. Elife.

[91] Mendiburo M.J., Padeken J., Fülöp S., Schepers A., Heun P. (2011). Drosophila CENH3 is sufficient for centromere formation. Science.

[92] Chen C.C., Dechassa M.L., Bettini E., Ledoux M.B., Belisario C., Heun P., Luger K., Mellone B.G. (2014). CAL1 is the Drosophila CENP-A assembly factor. J. Cell Biol..

[93] Chen C.C., Browers S., Lipinszki Z., Palladino J., Trusiak S., Bettini E., Rosin L., Przewloka M.R., Glover D.M., O’Neill R.J. (2015). Establishment of centromeric chromatin by the CENP-A assembly factor CAL1 requires FACT-mediated transcription. Dev. Cell.

[94] Jin C., Felsenfeld G. (2007). Nucleosome stability mediated by histone variants H3.3 and H2A.Z. Genes Dev..

[95] Gassmann R., Rechtsteiner A., Yuen K.W., Muroyama A., Egelhofer T., Gaydos L., Barron F., Maddox P., Essex A., Monen J. (2012). An inverse relationship to germline transcription defines centromeric chromatin in *C. elegans*. Nature.

[96] Steiner F.A., Henikoff S. (2014). Holocentromeres are dispersed point centromeres localized at transcription factor hotspots. Elife.

[97] Dunleavy E.M., Almouzni G., Karpen G.H. (2011). H3.3 is deposited at centromeres in S phase as a placeholder for newly assembled CENP-A in G1 phase. Nucleus.

[98] Monen J., Hattersley N., Muroyama A., Stevens D., Oegema K., Desai A. (2015). Separase cleaves the *N*-Tail of the CENP-A related protein CPAR-1 at the meiosis I metaphase-anaphase transition in *C. elegans*. PLoS ONE.

[99] Neumann P., Navrátilová A., Schroeder-Reiter E., Koblížková A., Steinbauerová V., Chocholová E., Novák P., Wanner G., Macas J. (2012). Stretching the rules: Monocentric chromosomes with multiple centromere domains. PLoS Genet..

[100] Neumann P., Pavlíková Z., Koblížková A., Fuková I., Jedličková V., Novák P., Macas J. (2015). Centromeres off the hook: Massive changes in centromere size and structure following duplication of CenH3 gene in *Fabeae* species. Mol. Biol. Evol..

[101] Hu Z., Huang G., Sadanandam A., Gu S., Lenburg M.E., Pai M., Bayani N., Blakely E.A., Gray J.W., Mao J.H. (2010). The expression level of HJURP has an independent prognostic impact and predicts the sensitivity to radiotherapy in breast cancer. Breast Cancer Res..

[102] Valente V., Serafim R.B., de Oliveira L.C., Adorni F.S., Torrieri R., Tirapelli D.P., Espreafico E.M., Oba-Shinjo S.M., Marie S.K., Paçó-Larson M.L. (2013). Modulation of HJURP (Holliday Junction-Recognizing Protein) levels is correlated with glioblastoma cells survival. PLoS ONE.

[103] De Tayrac M., Saikali S., Aubry M., Bellaud P., Boniface R., Quillien V., Mosser J. (2013). Prognostic significance of EDN/RB, HJURP, p60/CAF-1 and PDLI4, four new markers in high-grade gliomas. PLoS ONE.

[104] Montes de Oca R., Gurard-Levin Z.A., Berger F., Rehman H., Martel E., Corpet A., de Koning L., Vassias I., Wilson L.O., Meseure D. (2015). The histone chaperone HJURP is a new independent prognostic marker for luminal a breast carcinoma. Mol. Oncol..

[105] Tan E.H., Henry I.M., Ravi M., Bradnam K.R., Mandakova T., Marimuthu M.P., Korf I., Lysak M.A., Comai L., Chan S.W. (2015). Catastrophic chromosomal restructuring during genome elimination in plants. Elife.

[106] McAinsh A.D., Meraldi P. (2011). The CCAN complex: Linking centromere specification to control of kinetochore-microtubule dynamics. Semin. Cell Dev. Biol..

[107] Fujita R., Otake K., Arimura Y., Horikoshi N., Miya Y., Shiga T., Osakabe A., Tachiwana H., Ohzeki J., Larionov V. (2015). Stable complex formation of CENP-B with the CENP-A nucleosome. Nucleic Acids Res..

[108] Falk S.J., Guo L.Y., Sekulic N., Smoak E.M., Mani T., Logsdon G.A., Gupta K., Jansen L.E., van Duyne G.D., Vinogradov S.A. (2015). Chromosomes. CENP-C reshapes and stabilizes CENP-A nucleosomes at the centromere. Science.

[109] Kapoor M., de Oca Luna R.M., Liu G., Lozano G., Cummings C., Mancini M., Ouspenski I., Brinkley B.R., May G.S. (1998). The cenpB gene is not essential in mice. Chromosoma.

[110] Hudson D.F., Fowler K.J., Earle E., Saffery R., Kalitsis P., Trowell H., Hill J., Wreford N.G., de Kretser D.M., Cancilla M.R. (1998). Centromere protein B null mice are mitotically and meiotically normal but have lower body and testis weights. J. Cell Biol..

[111] Okada T., Ohzeki J., Nakano M., Yoda K., Brinkley W.R., Larionov V., Masumoto H. (2007). CENP-B controls centromere formation depending on the chromatin context. Cell.

[112] Marshall O.J., Choo K.H. (2012). Putative CENP-B paralogues are not present at mammalian centromeres. Chromosoma.

[113] Schueler M.G., Swanson W., Thomas P.J., Green E.D. (2010). Adaptive evolution of foundation kinetochore proteins in primates. Mol. Biol. Evol..

[114] Au W.C., Dawson A.R., Rawson D.W., Taylor S.B., Baker R.E., Basrai M.A. (2013). A novel role of the N terminus of budding yeast histone H3 variant Cse4 in ubiquitin-mediated proteolysis. Genetics.

[115] Boeckmann L., Takahashi Y., Au W.C., Mishra P.K., Choy J.S., Dawson A.R., Szeto M.Y., Waybright T.J., Heger C., McAndrew C. (2013). Phosphorylation of centromeric histone H3 variant regulates chromosome segregation in *Saccharomyces cerevisiae*. Mol. Biol. Cell.

[116] Mishra P.K., Guo J., Dittman L.E., Haase J., Yeh E., Bloom K., Basrai M.A. (2015). Pat1 protects centromere-specific histone H3 variant Cse4 from Psh1-mediated ubiquitination. Mol. Biol. Cell.

[117] Arimura Y., Shirayama K., Horikoshi N., Fujita R., Taguchi H., Kagawa W., Fukagawa T., Almouzni G., Kurumizaka H. (2014). Crystal structure and stable property of the cancer-associated heterotypic nucleosome containing CENP-A and H3.3. Sci. Rep..

[118] Goldberg A.D., Banaszynski L.A., Noh K.M., Lewis P.W., Elsaesser S.J., Stadler S., Dewell S., Law M., Guo X., Li X. (2010). Distinct factors control histone variant H3.3 localization at specific genomic regions. Cell.

[119] Voon H.P., Hughes J.R., Rode C., de La Rosa-Velázquez I.A., Jenuwein T., Feil R., Higgs D.R., Gibbons R.J. (2015). ATRX plays a key role in maintaining silencing at interstitial heterochromatic loci and imprinted genes. Cell Rep..

[120] Banaszynski L.A., Wen D., Dewell S., Whitcomb S.J., Lin M., Diaz N., Elsässer S.J., Chapgier A., Goldberg A.D., Canaani E. (2013). Hira-dependent histone H3.3 deposition facilitates PRC2 recruitment at developmental loci in ES cells. Cell.

[121] Chow C.M., Georgiou A., Szutorisz H., Maia e Silva A., Pombo A., Barahona I., Dargelos E., Canzonetta C., Dillon N. (2005). Variant histone H3.3 marks promoters of transcriptionally active genes during mammalian cell division. EMBO Rep..

[122] Wirbelauer C., Bell O., Schübeler D. (2005). Variant histone H3.3 is deposited at sites of nucleosomal displacement throughout transcribed genes while active histone modifications show a promoter-proximal bias. Genes Dev..

[123] Subramanian V., Mazumder A., Surface L.E., Butty V.L., Fields P.A., Alwan A., Torrey L., Thai K.K., Levine S.S., Bathe M. (2013). H2A.Z acidic patch couples chromatin dynamics to regulation of gene expression programs during ESC differentiation. PLoS Genet..

[124] Xu Y., Ayrapetov M.K., Xu C., Gursoy-Yuzugullu O., Hu Y., Price B.D. (2012). Histone H2A.Z controls a critical chromatin remodeling step required for DNA double-strand break repair. Mol. Cell.

[125] Sharma U., Stefanova D., Holmes S.G. (2013). Histone variant H2A.Z functions in sister chromatid cohesion in *Saccharomyces cerevisiae*. Mol. Cell Biol..

[126] Bruce K., Myers F.A., Mantouvalou E., Lefevre P., Greaves I., Bonifer C., Tremethick D.J., Thorne A.W., Crane-Robinson C. (2005). The replacement histone H2A.Z in a hyperacetylated form is a feature of active genes in the chicken. Nucleic Acids Res..

[127] Valdés-Mora F., Song J.Z., Statham A.L., Strbenac D., Robinson M.D., Nair S.S., Patterson K.I., Tremethick D.J., Stirzaker C., Clark S.J. (2012). Acetylation of H2A.Z is a key epigenetic modification associated with gene deregulation and epigenetic remodeling in cancer. Genome Res..

[128] Teves S.S., Henikoff S. (2011). Heat shock reduces stalled RNA polymerase II and nucleosome turnover genome-wide. Genes Dev..

[129] Chen P., Wang Y., Li G. (2014). Dynamics of histone variant H3.3 and its coregulation with H2A.Z at enhancers and promoters. Nucleus.

[130] Thatcher T.H., Gorovsky M.A. (1994). Phylogenetic analysis of the core histones H2A, H2B, H3, and H4. Nucleic Acids Res..

[131] Baldi S., Becker P.B. (2013). The variant histone H2A.V of *Drosophila*—Three roles, two guises. Chromosoma.

[132] Vernì F., Cenci G. (2015). The *Drosophila* histone variant H2A.V works in concert with HP1 to promote kinetochore-driven microtubule formation. Cell Cycle.

[133] Van Daal A., White E.M., Elgin S.C., Gorovsky M.A. (1990). Conservation of intron position indicates separation of major and variant H2As is an early event in the evolution of eukaryotes. J. Mol. Evol..

[134] Liu X., Li B., Gorovsky M.A. (1996). Essential and nonessential histone H2A variants in *Tetrahymena thermophila*. Mol. Cell Biol..

[135] Faast R., Thonglairoam V., Schulz T.C., Beall J., Wells J.R., Taylor H., Matthaei K., Rathjen P.D., Tremethick D.J., Lyons I. (2001). Histone variant H2A.Z is required for early mammalian development. Curr. Biol..

[136] Wu R.S., Kohn K.W., Bonner W.M. (1981). Metabolism of ubiquitinated histones. J. Biol. Chem..

[137] Li B., Pattenden S.G., Lee D., Gutiérrez J., Chen J., Seidel C., Gerton J., Workman J.L. (2005). Preferential occupancy of histone variant H2AZ at inactive promoters influences local histone modifications and chromatin remodeling. Proc. Natl. Acad. Sci. USA.

[138] Raisner R.M., Hartley P.D., Meneghini M.D., Bao M.Z., Liu C.L., Schreiber S.L., Rando O.J., Madhani H.D. (2005). Histone variant H2A.Z marks the 5' ends of both active and inactive genes in euchromatin. Cell.

[139] Zhang H., Roberts D.N., Cairns B.R. (2005). Genome-wide dynamics of Htz1, a histone H2A variant that poises repressed/basal promoters for activation through histone loss. Cell.

[140] Ranjan A., Mizuguchi G., FitzGerald P.C., Wei D., Wang F., Huang Y., Luk E., Woodcock C.L., Wu C. (2013). Nucleosome-free region dominates histone acetylation in targeting SWR1 to promoters for H2A.Z replacement. Cell.

[141] Watanabe S., Radman-Livaja M., Rando O.J., Peterson C.L. (2013). A histone acetylation switch regulates H2A.Z deposition by the SWR-C remodeling enzyme. Science.

[142] Weber C.M., Ramachandran S., Henikoff S. (2014). Nucleosomes are context-specific, H2A.Z-modulated barriers to RNA polymerase. Mol. Cell.

[143] Obri A., Ouararhni K., Papin C., Diebold M.L., Padmanabhan K., Marek M., Stoll I., Roy L., Reilly P.T., Mak T.W. (2014). ANP32E is a histone chaperone that removes H2A.Z from chromatin. Nature.

[144] Jeronimo C., Watanabe S., Kaplan C.D., Peterson C.L., Robert F. (2015). The histone chaperones FACT and Spt6 restrict H2A.Z from intragenic locations. Mol. Cell.

[145] Nekrasov M., Amrichova J., Parker B.J., Soboleva T.A., Jack C., Williams R., Huttley G.A., Tremethick D.J. (2012). Histone H2A.Z inheritance during the cell cycle and its impact on promoter organization and dynamics. Nat. Struct. Mol. Biol..

[146] Ogiyama Y., Ohno Y., Kubota Y., Ishii K. (2013). Epigenetically induced paucity of histone H2A.Z stabilizes fission-yeast ecoptic centromeres. Nat. Struct. Mol. Biol..

[147] Rangasamy D., Greaves I., Tremethick D.J. (2004). RNA interference demonstrates a novel role for H2A.Z in chromosome segregation. Nat. Struct. Mol. Biol..

[148] Kim H.S., Vanoosthuyse V., Fillingham J., Roguev A., Watt S., Kislinger T., Treyer A., Carpenter L.R., Bennett C.S., Emili A. (2009). An acetylated form of histone H2A.Z regulates chromosome architecture in Schizosaccharomyces pombe. Nat. Struct. Mol. Biol..

[149] Costanzi C., Pehrson J.R. (1998). Histone macroH2A1 is concentrated in the inactive X chromosome of female mammals. Nature.

[150] Chakravarthy S., Gundimella S.K., Caron C., Perche P.Y., Pehrson J.R., Khochbin S., Luger K. (2005). Structural characterization of the histone variant macroH2A. Mol. Cell Biol..

[151] Aygün O., Mehta S., Grewal S.I. (2013). HDAC-mediated suppression of histone turnover promotes epigenetic stability of heterochromatin. Nat. Struct. Mol. Biol..

[152] Yelagandula R., Stroud H., Holec S., Zhou K., Feng S., Zhong X., Muthurajan U.M., Nie X., Kawashima T., Groth M. (2014). The histone variant H2A.W defines heterochromatin and promotes chromatin condensation in Arabidopsis. Cell.

[153] Kustatscher G., Hothorn M., Pugieux C., Scheffzek K., Ladurner A.G. (2005). Splicing regulates NAD metabolite binding to histone macroH2A. Nat. Struct. Mol. Biol..

[154] Timinszky G., Till S., Hassa P.O., Hothorn M., Kustatscher G., Nijmeijer B., Colombelli J., Altmeyer M., Stelzer E.H., Scheffzek K. (2009). A macrodomain-containing histone rearranges chromatin upon sensing PARP1 activation. Nat. Struct. Mol. Biol..

[155] Nusinow D.A., Sharp J.A., Morris A., Salas S., Plath K., Panning B. (2007). The histone domain of macroH2A1 contains several dispersed elements that are each sufficient to direct enrichment on the inactive X chromosome. J. Mol. Biol..

[156] Dantzer F., Santoro R. (2013). The expanding role of PARPs in the establishment and maintenance of heterochromatin. FEBS J..

[157] Chen H., Ruiz P.D., Novikov L., Casill A.D., Park J.W., Gamble M.J. (2014). MacroH2A1.1 and PARP-1 cooperate to regulate transcription by promoting CBP-mediated H2B acetylation. Nat. Struct. Mol. Biol..

[158] Lavigne M.D., Vatsellas G., Polyzos A., Mantouvalou E., Sianidis G., Maraziotis I., Agelopoulos M., Thanos D. (2015). Composite macroH2A/NRF-1 nucleosomes suppress noise and generate robustness in gene expression. Cell Rep..

[159] Zhang X., Li X., Marshall J.B., Zhong C.X., Dawe R.K. (2005). Phosphoserines on maize CENTROMERIC HISTONE H3 and histone H3 demarcate the centromere and pericentromere during chromosome segregation. Plant Cell.

[160] Sporn J.C., Kustatscher G., Hothorn T., Collado M., Serrano M., Muley T., Schnabel P., Ladurner A.G. (2009). Histone macroH2A isoforms predict the risk of lung cancer recurrence. Oncogene.

[161] Timinszky G., Ladurner A.G. (2014). PARP1 and CBP lose their footing in cancer. Nat. Struct. Mol. Biol..

[162] Kapoor A., Goldberg M.S., Cumberland L.K., Ratnakumar K., Segura M.F., Emanuel P.O., Menendez S., Vardabasso C., LeRoy G., Vidal C.I. (2010). The histone variant macroH2A suppresses melanoma progression through regulation of CDK8. Nature.

[163] Kummar S., Chen A., Parchment R.E., Kinders R.J., Ji J., Tomaszewski J.E., Doroshow J.H. (2012). Advances in using PARP inhibitors to treat cancer. BMC Med..

[164] Schiewer M.J., Goodwin J.F., Han S., Brenner J.C., Augello M.A., Dean J.L., Liu F., Planck J.L., Ravindranathan P., Chinnaiyan A.M. (2012). Dual roles of PARP-1 promote cancer growth and progression. Cancer Discov..

[165] Green A.R., Caracappa D., Benhasouna A.A., Alshareeda A., Nolan C.C., Macmillan R.D., Madhusudan S., Ellis I.O., Rakha E.A. (2015). Biological and clinical significance of PARP1 protein expression in breast cancer. Breast Cancer Res. Treat..

[166] Skene P.J., Henikoff S. (2013). Histone variants in pluripotency and disease. Development.

[167] Vardabasso C., Hasson D., Ratnakumar K., Chung C.Y., Duarte L.F., Bernstein E. (2014). Histone variants: Emerging players in cancer biology. Cell. Mol. Life Sci..

[168] Vassetzky Y.S., Hair A., Razin S.V. (2000). Rearrangement of chromatin domains in cancer and development. J. Cell. Biochem. Suppl..

[169] De la Espina S.M.D., Alverca E., Cuadrado A., Franca S. (2005). Organization of the genome and gene expression in a nuclear environment lacking histones and nucleosomes: The amazing dinoflagellates. Eur. J. Cell Biol..

[170] Chan Y.H., Kwok A.C., Tsang J.S., Wong J.T. (2006). Alveolata histone-like proteins have different evolutionary origins. J. Evol. Biol..

[171] Gornik S.G., Ford K.L., Mulhern T.D., Bacic A., McFadden G.I., Waller R.F. (2012). Loss of nucleosomal DNA condensation coincides with appearance of a novel nuclear protein in dinoflagellates. Curr. Biol..

[172] Sandman K., Reeve J.N. (2006). Archaeal histones and the origin of the histone fold. Curr. Opin. Microbiol..

[173] Pereira S.L., Grayling R.A., Lurz R., Reeve J.N. (1997). Archaeal nucleosomes. Proc. Natl. Acad. Sci. USA.

[174] Decanniere K., Babu A.M., Sandman K., Reeve J.N., Heinemann U. (2000). Crystal structures of recombinant histones HMfA and HMfB from the hyperthermophilic archaeon Methanothermus fervidus. J. Mol. Biol..

[175] Arents G., Moudrianakis E.N. (1995). The histone fold: A ubiquitous architectural motif utilized in DNA compaction and protein dimerization. Proc. Natl. Acad. Sci. USA.

[176] Koster M.J., Snel B., Timmers H.T. (2015). Genesis of chromatin and transcription dynamics in the origin of species. Cell.

[177] Nabeel-Shah S., Ashraf K., Pearlman R.E., Fillingham J. (2014). Molecular evolution of NASP and conserved histone H3/H4 transport pathway. BMC Evol. Biol..

[178] Zovkic I.B., Paulukaitis B.S., Day J.J., Etikala D.M., Sweatt J.D. (2014). Histone H2A.Z subunit exchange controls consolidation of recent and remote memory. Nature.

[179] Bacolla A., Wells R.D. (2004). Non-B DNA conformations, genomic rearrangements, and human disease. J. Biol. Chem..

[180] Wells R.D. (2009). Discovery of the role of non-B DNA structures in mutagenesis and human genomic disorders. J. Biol. Chem..

